# Supplementary material for: The Proliferation of Chang Liver Cells After Simulated Microgravity Induction
Source: Curr Issues Mol Biol. 2025 Feb 27;47(3):164. doi: 10.3390/cimb47030164 (PMC11941360; doi:10.3390/cimb47030164)
Supplement: Supplementary file 1 [file cimb-47-00164-s001.zip › cimb-3487797-supplementary.pdf]

Supplementary Materials

1. O.D. value

Table S1. The O.D. value was analyzed by experiment WST-1.

No.	24h		72h	
	Control	SMG	Control	SMG
1	1.774	1.481	3.905	3.604
2	1.767	1.502	3.91	3.592
3	1.721	1.591	3.812	3.654

2. The viability, apoptosis, and necrosis of CCL-13

Table S2. The viability, apoptosis, and necrosis of CCL-13 was analyzed by Flow cytometry system.

No.	24h				72h			
	Control		SMG		Control		SMG	
	Viability	Apoptosis & necrosis	Viability	Apoptosis & necrosis	Viability	Apoptosis & necrosis	Viability	Apoptosis & necrosis
1	97.86	2.14	91.57	8.43	97.88	2.12	96.69	3.31
2	97.58	2.42	91.94	8.06	97.61	2.39	96.53	3.47
3	97.81	2.19	90.77	9.23	97.09	2.91	96.38	3.62

3. Cell Cycle progression analyzed by Cytell Microscope

Table S3. The percentage of CCL-13 cells in cell cycle phases was analyzed by Cell Cyle App. of Cytell Microscope.

No.	24h				72h			
	Control		SMG		Control		SMG	
	G0/G1 (%)	S + G2/ M (%)	G0/G1 (%)	S + G2/ M (%)	G0/G1 (%)	S + G2/ M (%)	G0/G1 (%)	S + G2/ M (%)
1	53.6	36.03	56.0	33.55	48.9	44.15	53.1	41.69
2	52.2	38.31	53.5	33.34	50.5	43.29	53.9	41.04
3	48.5	42.96	52.7	34.91	50.7	43.33	53.7	41.25
4	52.6	38.88	57.3	32.66	49.4	44.17	53.2	41.89
5	51.1	38.27	54.3	33.09	48.7	45.38	50.9	43.36
